# VEGF Spliced Variants: Possible Role of Anti-Angiogenesis Therapy

**DOI:** 10.1155/2012/162692

**Published:** 2011-10-13

**Authors:** Caroline Hilmi, Mélanie Guyot, Gilles Pagès

**Affiliations:** University of Nice Sophia Antipolis, Institute of Development and Cancer Research, UMR CNRS 6543, Centre Antoine Lacassagne, 33 Avenue de Valombrose, 06189 Nice, France

## Abstract

Angiogenesis has been targeted in retinopathies, psoriasis, and a variety of cancers (colon, breast, lung, and kidney). Among these tumour types, clear cell renal cell carcinomas (RCCs) are the most vascularized tumours due to mutations of the von Hippel Lindau gene resulting in HIF-1 alpha stabilisation and overexpression of Vascular Endothelial Growth Factor (VEGF). Surgical nephrectomy remains the most efficient curative treatment for patients with noninvasive disease, while VEGF targeting has resulted in varying degrees of success for treating metastatic disease. VEGF pre-mRNA undergoes alternative splicing generating pro-angiogenic isoforms. However, the recent identification of novel splice variants of VEGF with anti-angiogenic properties has provided some insight for the lack of current treatment efficacy. Here we discuss an explanation for the relapse to anti-angiogenesis treatment as being due to either an initial or acquired resistance to the therapy. We also discuss targeting angiogenesis via SR (serine/arginine-rich) proteins implicated in VEGF splicing.

## 1. Introduction

Therapies targeting angiogenesis seek to either decrease VEGF levels or to block its receptors resulting in the inhibition of downstream signalling pathways such as RAS/RAF/MEK/ERK and PI3 Kinase. Thus, molecules used in the clinic block VEGF or inhibit the tyrosine kinase activity of the VEGF receptors. These classical strategies evidently target endothelial cells and thus prevent angiogenesis but may also inhibit autocrine proliferative/survival pathways due to abnormal expression of VEGF receptors by tumour cells of different origins [1–9].

The main treatment commonly used is Bevacizumab (BVZ), a humanized IgG1 monoclonal antibody against VEGF [10]. A phase II clinical trial has shown that BVZ can significantly prolong the time to progression of disease in patients with metastatic renal-cell cancer [11]. However, only the BVZ plus interferon alpha (IFN) treatment has obtained approval by the Food and Drugs administration (FDA) in the United States of America and the European Medicines Agency (EMA) in Europe following phase III clinical assays [12, 13]. These clinical assays have demonstrated an increase in progression-free survival associated with the treatment combining IFN and BVZ compared to IFN alone. Unfortunately, BVZ plus IFN did not improve overall survival when compared to IFN monotherapy [14, 15]. Other treatments targeting the different VEGF receptors are Receptor Tyrosine Kinase Inhibitors (RTKI) such as sunitinib targeting VEGFR2, PDGFR, FLT3, and c-Kit or sorafenib targeting B-Raf, c-Raf, VEGFR2/3, PDGFR, FLT3, and c-Kit. These compounds are used in cases of advanced RCC with good or intermediate prognosis. Two clinical trials showed the benefit of using sunitinib for treating advanced RCC with a greater decrease in tumour size, an increase of progression-free survival of about nine months, and a better quality of life [16, 17]. Another phase III clinical trial has also demonstrated efficacy of sorafenib on RCC [17]. However, as for the BVZ plus IFN combined treatment, sunitinib or sorafenib did not increase overall survival of RCC patients. Axitinib [18] and pazopanib [19] are new VEGFR-TKI compounds generated for the treatment of RCC but it is too early to evaluate their efficacy compared to sorafenib or sunitinib. The other class of compounds targets the mTOR pathway. Patients who progressed on sorafenib or sunitinib as well as patients who have a poor prognosis are treated with mTOR blockers such as temsirolimus [20, 21] or everolimus [22]. Deforolimus is also a new generation of anti-mTOR compounds for the treatment of RCC [23].

## 2. Lack of Predictive Factors for the Success/Failure of Anti-Angiogenic Therapies

One of the main problems for patients treated with anti-angiogenic therapies is the lack of an effective predictive biomarker for their use. Many trials have tried to identify predictive biomarkers to assist in the selection of appropriate therapies. However, contradictory results from a number of these studies necessitate further investigations. Indeed, the circulating VEGF, thought to be associated with BVZ efficiency, is not predictive on benefit as has been shown in a phase II trial on RCC [11, 24]. Plasma levels of VEGF were not predictive of response to treatment by BVZ in other cancers either [24–26]. In the same way, no correlation had been observed with circulating endothelial cells (CECs) or circulating endothelial progenitors (CEPs) at only early stage, where RCC is rare to be diagnosed [27]. In summary, identification of predictive biomarkers for this treatment failed even though hypertension is thought to be a good candidate as a predictive marker of outcome with BVZ plus INF as the first-line treatment in advanced RCC [15, 28] as well as sunitinib in metastatic RCC treated patients [29].

## 3. Different Biological Effects Depending on Ligands and/or Receptors Involved

VEGF binds to its receptors, which then form either homodimers or heterodimers. Following the dimerization, the receptors are transphosphorylated and the downstream signalling pathways are activated. Furthermore, the kinase domain of each type of receptor is not the same and consequently, signalling will differ depending on the receptor involved. Thus, in the case of heterodimerization, the kinase domain of VEGFR1 will phosphorylate different tyrosine sites than VEGFR2 for example [30]. The same observations are also found with VEGFR2, and VEGFR3 [31]. Furthermore, depending on the ligand bound to the receptor, the signalling pathway can be rather different as has been well-described previously by Autiero et al. [32]. The situation is also complicated by the fact that neuropilin-1 a co-VEGFR is overexpressed on RCC cells and induces a specific activation of the PI3 Kinase pathway [33]. Whereas VEGFR are not expressed on RCC cells, neuropilin overexpression mediates potent proliferative and invasive capacities.

## 4. Implication of VEGFxxxb Isoforms

**Figure 1:**
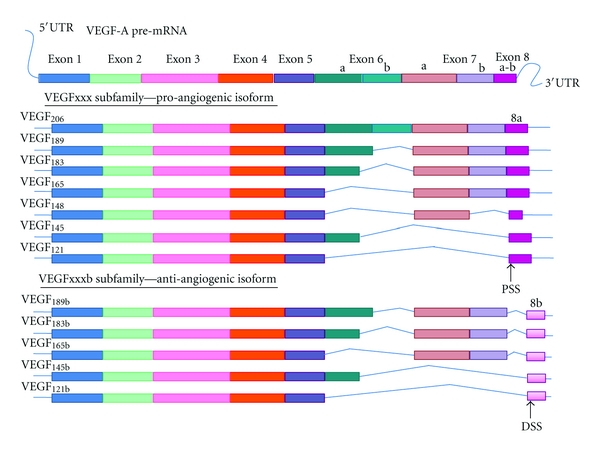
Alternative splicing of VEGF-A pre-mRNA. The pre-mRNA of VEGF-A undergoes alternative splicing leading to pro-angiogenic isoforms notated with the number of amino acids and containing as last exon, the exon 8a stemming from the Proximal Splicing Site (PSS) located at the beginning of exon 8. The more recent subfamily of VEGF isoforms containing five members so far, are anti-angiogenic and contain as last exon, the exon 8b resulting of the splicing at the Distal Splicing Site (DSS) located after the exon 8a.

VEGF-A is the first form of VEGF that was described twenty years ago for which the codiscoverer Napoleone Ferrara was awarded the Lasker Prize [34]. The pre-mRNA of VEGF-A undergoes alternative splicing leading to different isoforms noted as VEGFxxx (xxx stands for the number of amino acids present in proteins minus the signal peptide). The major ones are VEGF165, VEGF189, and VEGF121. There are also a few minor isoforms spliced from the pre-mRNA, which are VEGF206, VEGF183, VEGF145 and VEGF148, and VEGF111 although their functions remain less clear (Figure 1) [35–40].

In 2002 Bates et al. identified a splice variant of VEGF165, VEGF165b that is expressed in most normal tissues and downregulated in cancers especially in RCC [41]. Furthermore, this finding could put a full stop to the paradox of a high level of VEGF in podocytes where angiogenesis is not upregulated. As suggested by Bates and Harper, the codiscoverers of VEGFxxxb, the existence of anti-angiogenic forms of VEGF “needs reinterpretation or at worst, require repeating the experiment with reagent that differentiate between isoforms families” [42]. In light of the discovery of Bates et al., these forms of VEGF may be anti-angiogenic forms [41, 43]. After the identification of VEGF165b, a new sub-family of VEGFxxxb isoforms were identified (VEGF189b, VEGF121b) (Figure 1) [42]. 

Since then, a few publications assessed the anti-angiogenic or at least a less angiogenic outcome of VEGFxxxb isoforms by, in particular, the downregulation of VEGFR signalling pathway and a decrease of tumour growth [44–46]. These results have been achieved *in vitro* on proliferation and migration of endothelial cells along with *in vivo* studies on tumour volume of RCC, prostate, melanoma, and colorectal cancers and on experimental choroidal neo-vascularization [44, 47–50]. Moreover, the downregulation of VEGF165b expression leads to metastatic melanoma while VEGF165b expression prevents metastasis of malignant melanoma [51]. We can hypothesize that the ratio between the pro- and the anti-angiogenic or the less angiogenic isoforms may be crucial for the angiogenic balance. Recent results strongly suggest that VEGFxxxb may act as an anticancer therapy [45, 47, 48] and as an efficient therapy of eye pathologies associated with exacerbated angiogenesis [52].

## 5. VEGFxxxb Isoforms as an Explanation for the Failure of Anti-Angiogenic Treatments

The identification of VEGFxxxb isoforms has complicated the initial notion that targeting the pro-angiogenic forms of VEGF would be beneficial for the treatment of diseases associated with abnormal angiogenesis. Therefore, BVZ can recognize and bind VEGFxxxb as well as VEGFxxx isoforms since BVZ recognition motif is located in VEGFR-binding domain of VEGF [53]. Hence, the concomitant presence of VEGFxxx and VEGFxxxb isoforms may contribute to the inhibition of the anti-angiogenic effect of BVZ on tumour growth [45]. This hypothesis is particularly striking since we have detected VEGFxxx and VEGFxxxb isoforms in 70% of the RCC we have tested (Grépin, R and Pagès, G personal communication). Thus, BVZ can trap the VEGFxxxb isoforms that are, by themselves, capable of decreasing the activation of the angiogenic pathway. Effectively, VEGFxxxb homodimers bind to the VEGF receptors and block the downstream signalling pathway [44]. On the other hand, VEGFxxxb can heterodimerize with VEGF preventing VEGF-mediated activation of VEGF receptors [42]. Consequently, the presence of VEGFxxxb may have a double anti-angiogenic action through (i) direct downregulation of VEGF receptors signalling pathways and (ii) by inhibiting VEGF activation of the pathway. This finding could explain why patients treated with BVZ do not have as good results as expected if VEGFxxxb isoforms are still present.

Furthermore, treatment of breast, lung, colon, and kidney cancers has combined BVZ to conventional chemotherapies. A phase III trial of metastatic breast cancers, at primary diagnosis treated with BVZ plus Paclitaxel, showed a better benefit on progression-free survival than Paclitaxel alone [54]. Similarly, combination of BVZ plus Paclitaxel-Carboplatin in a randomized study of non-small-cell lung cancer showed a better benefit than Paclitaxel-Carboplatin alone [55]. Also, the addition of BVZ to Irinotecan, Fluorouracil and Leucovorin for treatment of metastatic colorectal cancer improved survival [56]. Finally, a randomized phase III trial of metastatic RCC showed the improvement on progression-free survival of the addition of BVZ to IFN as first-line treatment [12]. However, the relapse to therapy or an acquired resistance may be explained by either the redundancy of angiogenic factors or the selection of aggressive cells, showing the limit of these treatments and the necessity to switch to RTKI to bypass resistance to anti-VEGF therapy. We can hypothesize that the chemotherapeutic agents used in combination with BVZ may normalize the VEGF/VEGFxxxb ratio in favour of VEGFxxxb. Hence, targeting the “good and bad” VEGF isoforms may lead to selection of more aggressive cells rendering the therapeutic combination totally inefficient and even deleterious. Thus, in order to get the expected benefit, two different strategies could be investigated: either targeting specifically the pro-angiogenic VEGF isoform or treating patients with BVZ only in cases where the VEGFxxxb isoforms are absent, although it represents a third of patients as described above.

## 6. Regulation of VEGF Splicing Leading to Targeting Splicing for Therapy

**Figure 2:**
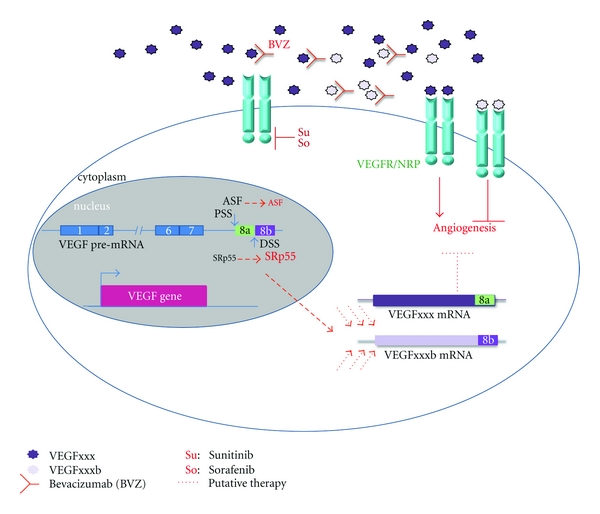
Existing and putative therapies targeting angiogenesis in RCC. VEGFxxx forms homodimers and interacts with their receptors inducing the activation of signalling pathways leading to increased angiogenesis. Homodimers of VEGFxxxb can bind the receptors blocking angiogenesis. However, the existence and function of potential VEGFxxx/VEGFxxxb heterodimers remain unclear. The monoclonal antibody Bevacizumab targets VEGFxxx blocking the VEGFR signalling pathways but also interacts with VEGFxxxb. Sorafenib and Sunitinib are Receptor Tyrosine Kinase inhibitors that interact with the kinase domain of VEGFR and thus inactivate the downstream signalling pathways. The VEGF gene is transcribed into a pre-mRNA that undergoes different splicing events leading to different isoforms. Splicing at Exon 8 will determinate the pro- or anti-angiogenic property of the produced protein. Hence, the use of the proximal splicing site (PSS) by in particular the splicing factor ASF/SF2 leads to pro-angiogenic -VEGFxxx- forms while the use of the distal splicing site (DSS) by another SR protein, SRp55 provides the anti-angiogenic -VEGFxxxb- forms. Combining all of this knowledge leads to propose that by acting on the VEGFxxx/VEGFxxxb ratio through the modulation of splicing, we could modify the angiogenic potential.

The study of VEGFxxxb isoforms regulation may be key for targeting the angiogenic balance in cancers and other pathologies. Most genes, like VEGF, can produce different transcripts, which result in the production of different protein isoforms. This phenomenon, known as alternative splicing, is mainly regulated by SR proteins. One of these proteins, ASF/SF2, has been described as a protooncogene with it being sufficient to induce cell transformation [57]. These observations provide a link between angiogenesis, cancer, and splicing. The study of VEGF splicing has illustrated in particular the need of SR proteins such as ASF/SF2 and SRp55. ASF/SF2, which is upregulated in most tumour types [58–60], favours the production of the pro-angiogenic isoforms, while SRp55 favours the production of the anti-angiogenic isoforms in normal cells [58]. Furthermore, TGF*β* treatment leads to an increase in the VEGFxxxb expression through an increase of SRp55 by the activation of the p38MAPK pathway. In contrast, IGF-1 stimulation leads to an increase in activated ASF/SF2 by phosphorylation and thus an increase of VEGFxxx isoforms [58]. All of the SR proteins are mainly regulated by SRPK and Clk kinases. Depending on the type of SR proteins, their phosphorylation can be mediated by specific kinases. Thus, the IGF-1-dependent increase of VEGFxxx could be blocked by the use of specific inhibitors of SRPK and/or Clk kinases. Thereby, TG0003 mainly inhibits Clk kinases and SRPIN340 inhibiting SRPK kinase, mainly involved in ASF/SF2 activation [60]. Furthermore, chromatin structure and associated modifications may influence the splicing [61]. The balance of splicing and transcription regulation depends on several other contributing factors, such as recruitment of RNA recognition, motif-containing proteins, or potential associated cofactors [62]. In addition, the sequence and the length of introns and exons play a major role for the splicing and the following translation of the mRNA. The use of Histone Deacetylase (HDAC) inhibitors such as sodium butyrate has been shown to promote the production of specific splice variants from a single pre-mRNA [63]. In the same manner, treatment of human lung microvascular endothelial cells (HLMECs) with sodium butyrate showed an increase of anti-angiogenic isoforms of VEGF suggesting that the treatment may act via a change of splicing factors acting on the balance of pro- and anti-angiogenic isoforms [64]. Thus, treatments already used in the clinic such as sunitinib or sorafenib may act through the same mechanisms leading to an increase of VEGFxxxb isoforms. Furthermore, when expressed, VEGFxxxb may be sequestered within the cytoplasm rather than secreted suggesting an intracellular role for these isoforms [65] as was already demonstrated for VEGF [3]. We can then predict that the combination of anti-angiogenic therapies and targeting of SRPK activity to alter the VEGF/VEGFxxxb balance may improve actual therapies and be a key to increased treatment efficiency (Figure 2).

##  Authors' Contribution

C. Hilmi and M. Guyot equally contributed to the paper.
